# Concomitant Statins and the Survival of Patients with Non-Small-Cell Lung Cancer Treated with Immune Checkpoint Inhibitors: A Meta-Analysis

**DOI:** 10.1155/2022/3429462

**Published:** 2022-07-05

**Authors:** Lei Zhang, Hong Wang, Jizheng Tian, Lili Sui, Xiaoyan Chen

**Affiliations:** Department of Oncology, The Hospital of Shunyi District of Beijing, Beijing 101300, China

## Abstract

Statins are suggested to improve cancer survival by possible anti-inflammatory effect. However, it remains unclear if concomitant use of statins could improve the efficacy of immune checkpoint inhibitors (ICIs) in patients with non-small-cell lung cancer (NSCLC). Accordingly, a meta-analysis was performed to systematically evaluate the effect of concomitant statins in NSCLC patients receiving ICIs. Relevant studies were obtained by literature search in PubMed, Embase, and Web of Science databases. A conservative random-effect model was used to combine the results. Eight cohorts including 2382 patients were included. The programmed death-1/ligand-1 inhibitors were used in seven studies; while the cytotoxic T-lymphocyte-associated protein 4 inhibitors were used in the other study. It was shown that concomitant use of statin did not significantly affect the progression-free survival (PFS, hazard ratio (HR): 0.86, 95% confidence interval (CI): 0.70 to 1.07, *P*=0.17; *I*^2^ = 62%) or overall survival (OS, HR: 0.86, 95% CI: 0.74 to 1.01, *P*=0.07; *I*^2^ = 29%) of NSCLC patients receiving ICIs. Subgroup analyses showed consistent results in studies with univariate or multivariate analytic models (*P* for subgroup analysis = 0.97 and 0.38 for the outcome of PFS and OS, respectively). In conclusion, concomitant use of statin seemed to have no significant influence on the survival of patients with NSCLC who were treated with ICIs.

## 1. Introduction

Immune checkpoint inhibitors (ICIs) are a group of novel anticancer agents which showed substantial therapeutic efficacy in patients with various solid cancers, including patients with non-small-cell lung cancer (NSCLC) [1–3]. Current ICIs primarily include the programmed death-1/ligand-1 (PD-1/PD-L1) inhibitors and cytotoxic T-lymphocyte associated protein 4 (CTLA4) inhibitors, which were shown to confer superior efficacy and less side effects as compared to conventional chemotherapy in patients with NSCLC [4, 5]. For patients with resectable NSCLC, a recent meta-analysis showed that neoadjuvant immunotherapy was feasible and safe, with major pathological response achieved in 52% of the patients and a low 30-day mortality of 0.6% [6]. The other systematic review and meta-analysis with 66 real-world studies confirmed the benefit of ICIs on survival in patients with pretreated and advanced NSCLC [7]. In view of the promising results of clinical trials, it could be anticipated that the use of ICIs in cancer will be increasing in patients with NSCLC [8]. However, previous studies also showed that therapeutic responses to ICIs varied among patients with cancer [9, 10]. For example, it has been shown that differences in age group, smoking history, metastasis status/site, and region may modify the potency of PD-1 inhibitors for the treatment of NSCLC [11]. Besides, concurrent medications have also been suggested to affect the therapeutic efficacy of ICIs in patients with various cancers [12]. Therefore, identification of clinical factors which may affect the efficacy of ICIs in patients with NSCLC is important in real-world clinical practice to maximize the possible benefit of ICIs [13].

The 3-hydroxy-3-methylglutaryl CoA (HMG-CoA) reductase inhibitors, also named as statins, are well-applied lipid-lowering medications [14]. Statins are commonly prescribed for patients with atherosclerosis and cardiovascular diseases, and about one-fifth of cancer patients receiving ICIs are also on statins [15]. Accumulating evidence shows that statins may also exert anticancer efficacy via multiple pharmacological mechanisms such as antiproliferation, anti-inflammation, proapoptosis, and anti-invasion [16, 17]. Accordingly, previous meta-analyses of observational studies have shown that statin use may favorably affect the survival of patients with lung cancer [18, 19]. However, none of the included studies in these meta-analyses included NSCLC patients treated with ICIs. Emerging evidence shows that statins also confer immunomodulation efficacy, which may synergistically enhance the anticancer efficacy of ICIs [20, 21]. However, a recent preclinical study showed that statins may decrease the expression of PD-LI in multiple cancer cell lines, including lung cancer, which raised the concern that concomitant statin use may interfere with the anticancer efficacy of ICIs [22]. Accordingly, results of recent observational studies evaluating the influence of concomitant statin use on survival of NSCLC patients treated with ICIs were not consistent [15, 23–28]. Some studies suggested that concomitant statin may improve the survival of these patients [23, 25, 28], while others did not show a significant influence [15, 24, 26, 27, 29]. Therefore, this meta-analysis was conducted to systematically evaluate the effect of concomitant statins on the therapeutic efficacy of ICIs in patients with NSCLC.

## 2. Materials and Methods

The Meta-Analysis of Observational Studies in Epidemiology (MOOSE) [30] statement was followed in conceiving, conducting, and reporting the study, and the methodology of the meta-analysis was in accordance with the recommendations of the Cochrane's Handbook [31] guideline. This meta-analysis was registered at INPLASY (International Platform of Registered Systematic Review and Meta-Analysis Protocols) with the registration number of INPLASY202250110.

### 2.1. Literature Retrieval

Studies fitting to the aim of the meta-analysis were retrieved by electronic database search of PubMed, Embase, and Web of Science from the inception of the databases to May 11, 2022. A combined search term was used, which is shown in Supplementary Materials. Filters of human studies, full-length articles, and publication in English were applied in the database search. As a supplementation, we manually checked the citations of the relevant original and reviewed articles for possible studies of interest.

### 2.2. Study Selection

The PICOS criteria were used for study inclusion.

P (patients) denotes adult patient with NSCLC receiving ICIs, including PD-1/PD-L1 inhibitors, CTLA4 inhibitors, or their combination. No restriction was applied to the pathological types of NSCLC (squamous cell carcinoma, adenocarcinoma, or other types of NSCLC).

I (exposure) denotes patients with concomitant use of statins as evidenced by the medical charts or other medical records with no restrictions to the category, dosages, or durations of statin use.

C (control) denotes patients without concomitant use of statins which were also evidenced by the medical charts or other medical records.

O (outcomes) denotes progression-free survival (PFS) and/or overall survival (OS) between users and nonusers of statins, reported as relative risk. We defined OS as the time elapsed from treatment and to the date of death from any cause and PFS as the interval between initiation of the treatment and the first recurrence or progression event.

S (study design) denotes cohort studies published as full-length articles.

Reviews, preclinical studies, studies with non-NSCLC patients, studies with no treatment of ICIs, or studies that did not report the outcomes of interest were removed. Moreover, grey literature studies such as conferences, abstracts, or unpublished data were also not considered. These materials were generally not peer-reviewed, and inclusion of these data into the meta-analysis may confound the results.

### 2.3. Data Collection and Quality Assessment

Two independent authors conducted literature search and analysis, data collection, and study quality assessment separately. If discrepancies were encountered, the corresponding author joined the discussion for final judgement. Data of study information, patient demographic factors, types of ICIs, definition of concurrent statin application, outcomes reported, and analytic methods were collected. Study quality assessment was achieved via the Newcastle–Ottawa Scale [32], with scoring regarding the criteria for participant selection, comparability of the groups, and the validity of the outcomes. The scale ranged between 1 and 9 stars, with the larger number of stars presenting higher study quality.

### 2.4. Statistical Analyses

The main objective was to determine the influence of concomitant statin on survival of patients with NSCLC on treatment of ICIs, which was presented with hazard ratios (HRs) as well as their confidence intervals (CIs). Using the 95% CIs or *P* values, data of RRs and the standard errors (SEs) could be calculated, and a subsequent logarithmical transformation was conducted to keep stabilized variance and normalized distribution [31]. Between-study heterogeneity was estimated with Cochrane's *Q* test and the *I*^2^ statistic [33], with *I*^2^ > 50% reflecting the significant heterogeneity. A random-effect model was applied to combine the results by incorporating the influence of heterogeneity [31]. We observed the influence of each study on the overall results by performing sensitivity analyses, which omitted one study at a time [34]. Subgroup analyses were also performed to explore the different analytic model of the study on the outcome. By construction of the funnel plots, the publication bias was estimated based on the visual judgement of the symmetry of the plots, supplemented with Egger's regression asymmetry test [35]. The RevMan (Version 5.1; Cochrane Collaboration, Oxford, UK) software package was applied for these analyses.

## 3. Results

### 3.1. Studies Obtained

**Figure 1:**
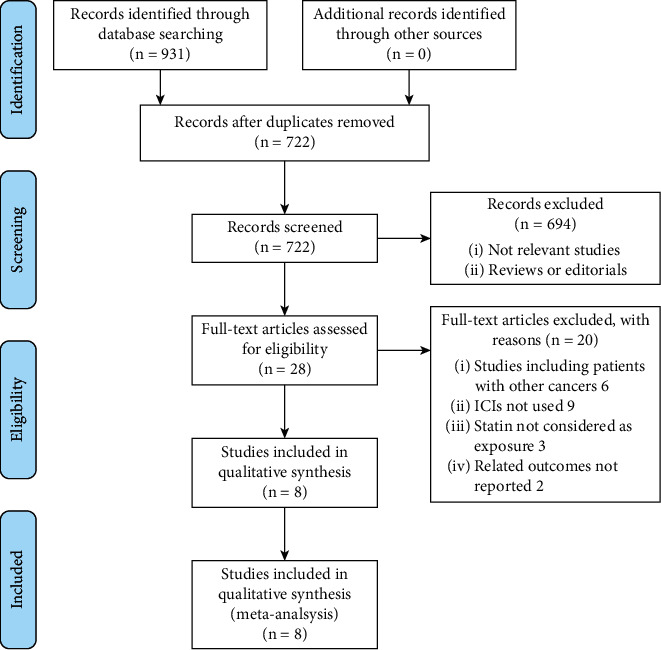
Summarized process of literature search and study retrieval.

Figure 1 shows the process of literature analysis. In short, the initial search of the databases retrieved 722 studies after removing the duplicated records. Then, an additional of 694 articles were excluded since the contents of titles and abstracts indicated they were not relevant to the meta-analysis, which made a total of 28 studies for a full-text review. Finally, after excluding 20 studies through full-text review, eight cohort studies [15, 23–29] were included. Reasons for removing the 19 studies are also presented in Figure 1.

### 3.2. Characteristics of the Included Studies

As shown in Table 1, eight cohort studies [15, 23–29] including 2382 patients with NSCLC who were receiving ICIs were included. These studies were performed within 2019 to 2022 and located in Japan [23, 27, 29], Czech [24], Italy [25, 26, 28], and France [15], respectively. Most of the studies were retrospective except one study [23], which was prospective. Seven of the studies [23–29] included advanced NSCLC patients treated with PD-1/PD-LI inhibitors, such as pembrolizumab, nivolumab, or atezolizumab etc., while the other study included patients treated with PD-1/PD-LI inhibitors, CTLA4 inhibitors, or their combination [15]. The mean ages of the patients were 65 to 71 years. Concomitant use of statin was validated by medical records or database in all studies. A total of 513 (21.5%) patients were with concomitant use of statin. Four studies reported the association between concomitant statin and survival outcomes with univariate analyses [15, 23, 26, 28], while the other four studies reported with multivariate analyses [24, 25, 27, 29]. Variables including demographic features, cancer characteristics, and smoking history were adjusted in the multivariate models. The NOS of the included studies were 6 to 8 stars, suggesting generally good study quality (Table 2).

### 3.3. Concomitant Statin and PFS in NSCLC Patients Taking ICIs

**Figure 2:**
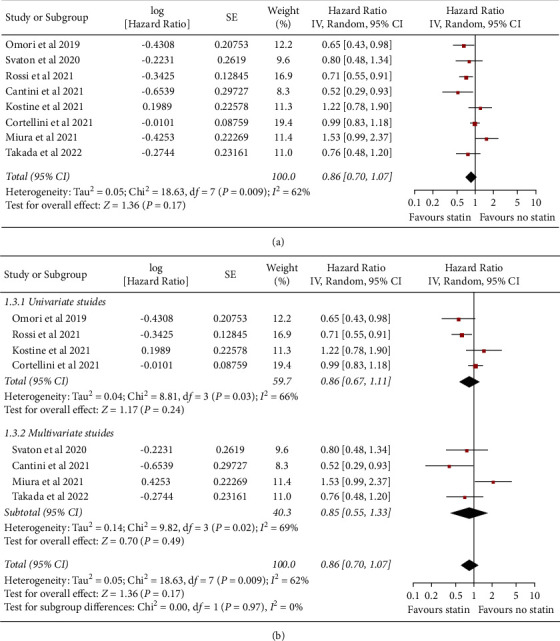
Forest plots for the meta-analysis of the influence of concomitant statin on PFS of NSCLC patients receiving ICIs. (a) Forest plots for the overall meta-analysis; (b) subgroup analysis in univariate and multivariate studies.

Influence of concomitant statin on PFS in NSCLC patients treated with ICIs was reported in all of the eight studies. Pooled results showed that concomitant use of statin was not associated with a significantly improved PFS (HR: 0.86, 95% CI: 0.70 to 1.07, *P*=0.17; Figure 2(a)) with significant heterogeneity (*P* for Cochrane's *Q* test = 0.009, *I*^2^ = 62%). Sensitivity analysis by excluding one study at a time showed consistent result (HR: 0.81 to 0.90, *P* all >0.05). Specifically, sensitivity analysis limited to studies of patients taking PD-1/PD-L1 only showed consistent result (HR: 0.83, 95% CI: 0.66 to 1.03, *P*=0.10, *I*^2^ = 64%). Subgroup analyses also showed consistent results in univariate (HR: 0.86, 95% CI: 0.67 to 1.11, *P*=0.24, *I*^2^ = 66%) and multivariate studies (HR: 0.85, 95% CI: 0.55 to 1.33, *P*=0.49, *I*^2^ = 69%; *P* for subgroup difference = 0.97, Figure 2(b)).

### 3.4. Concomitant Statin and OS in NSCLC Patients Taking ICIs

**Figure 3:**
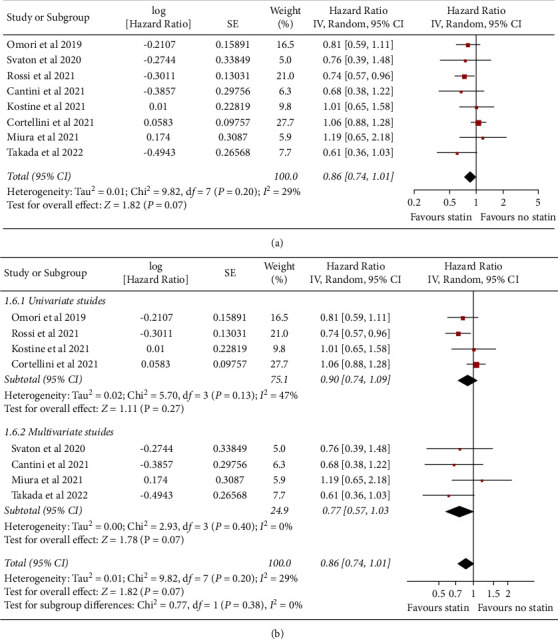
Forest plots for the meta-analysis of the influence of concomitant statin on OS of NSCLC patients receiving ICIs. (a) Forest plots for the overall meta-analysis; (b) subgroup analysis in univariate and multivariate studies.

Influence of concomitant statin on OS in NSCLC patients treated with ICIs was also reported in all of the eight studies. Pooled results showed that concomitant use of statin was not associated with a significantly improved OS (HR: 0.86, 95% CI: 0.74 to 1.01, *P*=0.07; Figure 3(a)) with mild heterogeneity (*P* for Cochrane's *Q* test = 0.20, *I*^2^ = 29%). Sensitivity analysis by excluding one study at a time showed consistent results (HR: 0.79 to 0.91, *P* all >0.05). Specifically, sensitivity analysis limited to studies of patients taking PD-1/PD-L1 only showed consistent results (HR: 0.84, 95% CI: 0.71 to 1.01, *P*=0.07, *I*^2^ = 37%). Subgroup analyses also showed consistent results in univariate (HR: 0.90, 95% CI: 0.74 to 1.09, *P*=0.27, *I*^2^ = 47%) and multivariate studies (HR: 0.77, 95% CI: 0.57 to 1.03, *P*=0.07, *I*^2^ = 0%; *P* for subgroup difference = 0.38, Figure 3(b)).

### 3.5. Publication Bias

**Figure 4:**
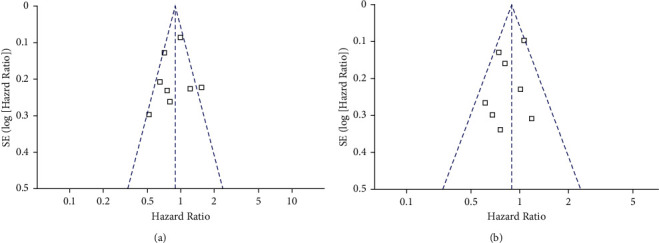
Funnel plots for the publication bias underlying the meta-analyses. (a) Funnel plots for the meta-analysis of PFS; (b) funnel plots for the meta-analysis of OS.

Figures 4(a) and 4(b) display the funnel plots for the outcomes of PFS and OS. Visual inspection revealed symmetry of the plots, reflecting a low risk of publication biases. Egger's regression tests also indicated low risk of publication biases (*P*=0.512 and 0.693, respectively).

## 4. Discussion

In this meta-analysis, we pooled the results of eight cohort studies and showed that concomitant use of statins in NSCLC patients receiving ICIs treatment was not associated with significantly improved survival outcomes, including PFS and OS. Further sensitivity analyses by excluding one study at a time did not significantly affect the results. Moreover, sensitivity analysis limited to studies including NSCLC patients receiving PD-1/PD-1L inhibitors also showed similar results. Finally, subgroup analyses showed that concomitant use of statins in NSCLC patients was not associated with a significantly improved PFS or OS in univariate or multivariate studies. Taken together, current evidence primarily based on retrospective studies in NSCLC patients taking PD-1/PD-1L inhibitors did not show that concurrent use of statins was associated with a significantly improved survival in these patients. Although these results should be further confirmed in large-scale prospective studies, these results did not support the previous hypothesis that concomitant statins may improve the anticancer efficacy of ICIs in patients with NSCLC.

To the best of our knowledge, this is the first meta-analysis which summarized the current evidence regarding the influence of concomitant statins in patients with NSCLC receiving ICIs treatment. During the preparation of the manuscript, a meta-analysis was published to evaluate the possible influence of concomitant medications, including statins, on the survival in patients with advanced cancer who were treated with ICIs [36]. However, only five studies were included in their analysis, and studies including various malignancies were included [36]. Although it was shown that concomitant statin may be associated with improved PFS and OS in overall meta-analyses including patients with advanced cancers, the authors were not able to determine whether the association remained in patients with NSCLC [36]. This is important because it has been acknowledged that patients with different malignancies may respond differently to immunotherapies [37]. Our meta-analysis, on the other hand, included eight studies of patients with NSCLC only and showed that concomitant use of statins in NSCLC patients was not associated with a significantly improved PFS or OS. The robustness of the finding was validated by sensitivity analyses and subgroup analyses in univariate and multivariate studies. Results of the meta-analysis were also not consistent with previous findings in preclinical studies which showed synergistic anticancer actions of statins on the anticancer efficacy of ICIs [20]. Previous experimental studies showed that statins may synergize with PD-1 inhibitors via attenuating the expression of PD-1 and CTLA-4 in T cells and increasing antigen occupation in dendritic cells [38, 39]. However, these benefits of statins were primarily observed in cultured cancer cell lines or murine models of cancer, which may not be the case in patients with advanced NSCLC, possibly due to the difference in dosages of statins and the influences of comorbidities and concurrent medications. Future studies are warranted to clarify the mechanisms underlying the inconsistency between related preclinical and observational studies.

Two early meta-analyses have suggested the possible benefit of statin use on survival of patients with lung cancer [18, 19]. However, it should be clarified that none of the studies which contributed to these meta-analyses include patients that received ICIs. Therefore, the previous suggested survival benefit of statins for lung cancer patients may not exist for NSCLC patients receiving ICIs. In fact, a recent retrospective cohort study including 2757 patients with advanced cancer who were treated with ICIs showed that concurrent statin therapy in these patients was independently associated with higher risk of skeletal myopathies [40]. The possible influences of concurrent statins on the prognosis and the possible risk of adverse events in NSCLC patients who were treated with ICIs should be further evaluated.

This meta-analysis also has some limitations. Firstly, most of the studies were retrospective and of limited sample sizes, results of which may be affected by possible recall and selection biases. Accordingly, large-scale prospective cohort studies are needed to validate the findings. Secondly, we could not determine whether difference in the individual category of statins may affect the association of interest since related data were rarely reported among the included studies. Studies are warranted in the future for investigation. Besides, most of the included patients received PD-1/PD-L1 inhibitors rather than CTLA-4 inhibitors. The possible influence of concomitant statin use on survival in NSCLC patients receiving CTLA-4 inhibitors is still to be determined. Finally, although subgroup analysis of multivariate analysis showed consistent results, it remained unknown whether age, sex, ethnicity, comorbidities, and other concurrent medications may significantly affect the association between concomitant statin and survival of these patients, which may be the source of heterogeneity among the included studies. Meta-analysis based on individual-patient data should be performed for further evaluation.

To sum up, current evidence from observational studies did not show that concurrent use of statins was associated with a significantly improved survival in NSCLC patients receiving ICIs. Although these results should be further confirmed in large-scale prospective studies, in view of the possible increased risk of adverse events such as skeletal myopathies, concomitant use of statins in NSCLC patients receiving ICIs should be cautious.

## Figures and Tables

**Table 1:** Characteristics of the included cohort studies.

Study	Country	Design	Patient characteristics	Sample size	Mean age (years)	Male (%)	ICIs used	Definition of statin use	Number of statin users	Outcomes reported	Variables adjusted
Omori et al. [23]	Japan	*P*	Advanced NSCLC	67	67	69	Nivolumab	Concomitant statin evidenced by the medical records	10	PFS and OS	None
Svaton et al. [24]	Czech	*R*	Advanced NSCLC	224	67	59	Nivolumab	Concomitant statin evidenced by the medical records	31	PFS and OS	Age, sex, PS, smoking, histologic type, cancer stage, and concurrent medications
Rossi et al. [28]	Italy	*R*	Metastatic NSCLC	122	71	65	Nivolumab, pembrolizumab, or atezolizumab	Concomitant statin evidenced by the medical records	70	PFS and OS	None
Cantini et al. [25]	Italy	*R*	Advanced NSCLC	179	67	72	Nivolumab or pembrolizumab	Concomitant statin evidenced by the medical records	39	PFS and OS	Age, sex, smoking, PS, and histologic type
Kostine et al. [15]	France	*R*	Advanced NSCLC	150	65	70	PD-1/PD-L1 and/or CTLA4 inhibitors	Concomitant statin evidenced by the medical records	32	PFS and OS	None
Cortellini et al. [26]	Italy	*R*	Metastatic NSCLC	950	70	66	Pembrolizumab	Concomitant statin evidenced by the medical records	252	PFS and OS	None
Miura et al. [27]	Japan	*R*	Metastatic NSCLC	300	65	75	Nivolumab or pembrolizumab	Concomitant statin evidenced by the medical records	26	PFS and OS	Age, sex, PS, histologic type, previous therapy, and concurrent medications
Takada et al. [29]	Japan	*R*	Advanced or recurrent NSCLC	390	67	79	Nivolumab or pembrolizumab	Concomitant statin evidenced by the medical records	53	PFS and OS	Age, sex, PS, cancer stage, histologic type, mutational status, and BMI

ICIs, immune checkpoint inhibitors; *P*, prospective; *R*, retrospective; NSCLC, nonsmall cell lung cancer; PD-1/PD-LI, programmed death-1/ligand-1; CTLA4, cytotoxic T-lymphocyte associated protein 4; PFS, progression-free survival; OS, overall survival; PS, performance status; BMI, body mass index.

**Table 2:** Details of study quality evaluation via the Newcastle–Ottawa Scale.

Study	Representativeness of the exposed cohort	Selection of the nonexposed cohort	Ascertainment of exposure	Outcome not present at baseline	Control for age	Control for other confounding factors	Assessment of outcome	Enough long follow-up duration	Adequacy of follow-up of cohorts	Total
Omori et al. [23]	1	1	1	1	0	0	1	1	1	7
Svaton et al. [24]	0	1	1	1	1	1	1	1	1	8
Rossi et al. [28]	0	1	1	1	0	0	1	1	1	6
Cantini et al. [25]	0	1	1	1	1	1	1	1	1	8
Kostine et al. [15]	0	1	1	1	0	0	1	1	1	6
Cortellini et al. [26]	0	1	1	1	0	0	1	1	1	6
Miura et al. [27]	0	1	1	1	1	1	1	1	1	8
Takada et al. [29]	0	1	1	1	1	1	1	1	1	8

## Data Availability

The data adopted in this meta-analysis are available from the corresponding author on reasonable request.

## Supplementary Materials

Supplementary Materials
*Database Search Strategy*. (“statin” OR “3-hydroxy-3-methyl-glutarylCoA reductase inhibitor” OR “CS-514” OR “statin” OR “simvastatin” OR “atorvastatin” OR “fluvastatin” OR “lovastatin” OR “rosuvastatin” OR “pravastatin” OR “pitavastatin”) AND “lung cancer” AND (“survival” OR “mortality” OR “prognosis” OR “death” OR “recurrence” OR “collapse”).
