# Supplementary material for: Concomitant Statins and the Survival of Patients with Non-Small-Cell Lung Cancer Treated with Immune Checkpoint Inhibitors: A Meta-Analysis
Source: Int J Clin Pract. 2022 Jul 5;2022:3429462. doi: 10.1155/2022/3429462 (PMC9276478; doi:10.1155/2022/3429462)
Supplement: Supplementary Materials — Database Search Strategy. (“statin” OR “3-hydroxy-3-methyl-glutarylCoA reductase inhibitor” OR “CS-514” OR “statin” OR “simvastatin” OR “atorvastatin” OR “fluvastatin” OR “lovastatin” OR “rosuvastatin” OR “pravastatin” OR “pitavastatin”) AND “lung cancer” AND (“survival” OR “mortality” OR “prognosis” OR “death” OR “recurrence” OR “collapse”). [file 3429462.f1.docx]

**Database search strategy**

("statin" OR "3-hydroxy-3-methyl-glutarylCoA reductase inhibitor" OR "CS-514" OR "statin" OR "simvastatin" OR "atorvastatin" OR "fluvastatin" OR "lovastatin" OR "rosuvastatin" OR "pravastatin" OR "pitavastatin") AND "lung cancer" AND ("survival" OR "mortality" OR "prognosis" OR "death" OR "recurrence" OR "collapse")
